# Psychometric properties of MyCommunication-Children: an item bank for measuring communicative participation in children with speech, language, voice disorders or hearing loss

**DOI:** 10.1186/s41687-026-01118-w

**Published:** 2026-06-17

**Authors:** Eline Alons, Margreet Roelien Luinge, Ellen Gerrits, Nicole Ter Wal, Lizet Van Ewijk, Benjamin Schalet, Caroline Barbara Terwee

**Affiliations:** 1 Research Group Speech and Language Therapy, HU University of Applied Sciences Utrecht, Utrecht, Netherlands; 2 Epidemiology and Data Science, Amsterdam UMC Location VUmc, Amsterdam, Netherlands; 3 Research Centre Healthy Ageing, Youth, Education, and Society, Hanze University of Applied Sciences, Groningen, Netherlands; 4 Department of Languages, Literature, and Communication; Institute for Language Sciences, Utrecht University, Utrecht, Netherlands; 5 Methodology, Amsterdam Public Health Research Institute, Amsterdam, Netherlands

**Keywords:** Children, Outcome, Structural validity, Item bank, Communication, Participation

## Abstract

**Background:**

Communicative participation is the most important outcome of speech and language therapy. However, there are no instruments to measure communicative participation in children. This paper focuses on assessing the psychometric properties of MyCommunication-Children, an item bank designed to measure communicative participation in children with speech, language, or voice disorders, as well as hearing loss.

**Methodology:**

A total of 310 children with communication difficulties participated in the study. They completed the full item pool of 49 items, along with three comparator questionnaires: the PROMIS® Pediatric Peer Relationships and two single items measuring communication and communicative participation. Psychometric evaluation focused on structural validity, construct validity, and reliability.

**Results:**

Seventeen of the 49 items were removed through systematic item reduction. Confirmatory factor analysis (CFA) and bifactor modeling supported sufficient unidimensionality, indicating that communicative participation can be reliably measured as a single construct. Moderate correlations with related constructs (e.g., communication, communicative participation, and peer relationships) further supported the construct validity of MyCommunication-Children. Two short forms were developed for different purposes: a 10-item version measuring communicative participation across all contexts, and a 9-item version measuring communicative participation within the school context. Reliability analyses showed high reliability for the full item bank and sufficient reliability for both short forms.

**Conclusion:**

The results provide sufficient evidence to support the validity and reliability of the MyCommunication-Children instrument for measuring communicative participation from the perspective of children with communication difficulties.

**Supplementary Information:**

The online version contains supplementary material available at 10.1186/s41687-026-01118-w.

## Introduction

The ability to communicate is essential for participation in daily life [1]. Communication problems, such as language disorders, speech disorders, voice disorders, or hearing loss, have impact on people’s participation in daily life. This concept is captured by the term *communicative participation*, which is defined as “taking part in life situations where knowledge, information, ideas or feelings are exchanged” [2]. Communicative participation is grounded in the World Health Organization’s International Classification of Functioning, Disability and Health (ICF) [3, 4]. Within the ICF, health is represented across several components, including (a) body structures and functions, (b) activities and participation, and (c) contextual factors. Communicative participation is situated within the activities and participation component. It refers to all situations in the ICF activities and participation component in which communication is required to participate meaningfully.

In recent years, communicative participation has gained increasing recognition as the most important outcome of speech and language therapy [5, 6]. This is reflected in the participation-focused framework proposed by Baylor and Darling-White [5], which places communicative participation at the core of intervention planning, with other components of the ICF organized around it to support more person-centered care. Although communicative participation is a key outcome of speech and language therapy [5–8], clinical assessments often focus on impairments at the level of body functions and structures and activities instead of participation [6, 9, 10]. A frequent cited reason for this is the limited availability of assessment tools specifically designed to evaluate communicative participation [6, 7, 11, 12].

Perceived participation difficulties are captured by patient-reported outcomes (PROs). Research suggests that PROs should be incorporated as primary indicators of restrictions in participation and to evaluate interventions [13–15]. PROs are measured by using Patient Reported Outcome Measures (PROMs). For adults, several PROMs measure communicative participation, such as the Communicative Participation Item Bank (CPIB) [16] and MyCommunication-Adults [17]. While these instruments are well-validated and conceptually robust for adults, they are not suitable for children, as children participate in different contexts [7]. For children, no PROMs that measure communicative participation are available [7, 18]. Existing proxy measures, such as the Focus on the Outcomes of Communication Under Six (FOCUS) [19] or instruments assessing broader aspects of communication and adaptive functioning (e.g., Vineland-3 [20], ABAS-3 [21], PEDI-CAT [22]), provide useful information through parent or clinician reports, but do not capture children’s actual participation in communicative situations from the child’s own perspective. Self-report should be enabled whenever possible, as children have a unique perspective on their own experiences [23, 24]. Discrepancies between parent and child reports may arise because agreement is higher for observable outcomes than for non-observable ones [25]. Communicative participation contains both observable and non-observable elements, of which only the child can provide a reliable perspective [26]. Although the existing proxy measures provide valuable information, there is an additional need for a PROM, capturing the child’s perspective.

To address this gap, the process of development of a new PROM that measures communicative participation in children, adolescents, and young adults was initiated. To ground the development of the new PROM in a clear conceptual framework, the construct of communicative participation was further operationalized in collaboration with the authors of MyCommunication-Adults [17]. The definition of Eadie and colleagues shows that communicative participation is a combination of two separate constructs: (1) communication (the exchange of knowledge, information, ideas, or feelings); and (2) participation (taking part in life situations). The operationalization entailed considerations for both communication and participation. In communication, we considered the “exchange” as “an exchange with the opportunity for a direct, prolonged or behavioral response between two communicative partners with the purpose of establishing joint meaning”. For participation, we chose to interpret “life situations” as “the performance of social roles with in the ICF Activities and Participation domains *mobility*,* self-care*,* domestic life*,* interpersonal interactions and relationships*,* major life areas* and *community*,* social and civic life*” (domain 4–9) [3].

The developmental process involved several steps of extensive research, in which the standards of the COnsensus-based Standards for the selection of health Measurement INstruments (COSMIN) initiative were used [27]. COSMIN provides internationally accepted guidelines for the development and validation of patient-reported outcome measures (PROMs), aiming to ensure that measurement instruments are conceptually sound, comprehensive, and psychometrically robust. In the COSMIN taxonomy, three quality domains are distinguished: reliability, validity, and responsiveness. Each domain comprises one or more measurement properties that describe different aspects of the quality of a measurement instrument. The developmental process started with establishing good content validity.

First, relevant, existing items that measure communicative participation were identified in a systematic review of PROMs and parent reports [18]. Hereafter, two concept elicitation studies were conducted that explored the perspectives of adolescents and young adults [28] and children [29] on communicative participation. In the first study, adolescents and young adults (*n* = 12, aged 13–27 years) with diverse communication disorders identified 234 self-experienced situations and 37 concepts influencing communicative participation, divided over six categories: person, location, topic, pace, moment, and mode. In the second study, children aged 5–11 years (*n* = 13) reported 171 situations and 44 concepts, reflecting the same overarching categories but including several child-specific concepts (e.g., consulting/negotiating with peers, losing turn when waiting). Both studies led to new items. Based on all identified items, preliminary versions of MyCommunication-Youth were developed, comprising separate versions tailored to school-related life stages of children (primary school), adolescents (secondary school), and young adults [30]. Developmental variability within these versions should be considered, as there may be differences in cognitive, social, and/or communicative abilities. Content validity was further evaluated through a focus group with professionals and two rounds of cognitive interviews, in which children, adolescents, and young adults assessed the comprehensibility, relevance, and comprehensiveness of the items [30]. These interviews confirmed that the school-based adaptation of items was appropriate across the age range [30], and that MyCommunication–Youth was considered a relevant, comprehensible, and comprehensive instrument for measuring communicative participation [30].

The next step in the development of MyCommunication-Youth is the assessment of the psychometric properties of the instrument. In this paper, the analysis of the version for children, MyCommunication-Children is described. The aim is to work towards an item bank, by using item response theory (IRT) [31], a modern measurement approach that positions both items and respondents on the same underlying scale. This placement of items on the scale is possible with estimated item parameters, derived from the IRT model. Once obtained, these item parameters from a single large item bank can be used to construct multiple PROMs, tailored to specific populations, or levels of ability. In addition, IRT-calibrated item banks allow for computer adaptive testing (CAT), in which the test adapts based on the previously answered item. This way, items are tailored to the ability of the individual.

The aim of this study is to assess the psychometric properties of MyCommunication-Children, by assessing the structural validity, construct validity and reliability of the instrument. In addition, short forms will be proposed to use in clinical practice in the general population of children with communication problems, in situations where CAT is not feasible.

## Method

### Participants

Children in primary school from the age of 7 years old with speech-, language- or voice disorders and/or hearing loss were recruited. However, in cases where speech and language therapists judged that younger children (aged 4–6) were cognitively able to complete the questionnaire, they were also included. Eligibility was based on attendance in primary education (in the Dutch context, children typically enter primary education at the age of four) rather than a strict upper age limit; consequently, no age-specific upper boundary was defined. We aimed for a diverse sample in terms of age and type of communication problem, with the following exclusion criteria: (1) children with severe multiple disabilities who did not live at home were excluded, as the content of the PROM does not align with their daily lives; (2) children with cognitive impairments, based on the professional judgement of SLT, as this population was not part of the study on content validity.

### Ethics approval and consent to participate

The study was approved by the Internal Review Board of the HU University of Applied Sciences (reference number 303-000-2024) and was conducted according to the principles of the Declaration of Helsinki and in accordance with the Dutch Medical Research Involving Human Subjects Act (WMO).

Participants and their parents were informed with an informed consent letter and an optional video for participants with language or reading difficulties. For children under the age of 12, consent was provided by a parent or legal guardian. Children aged 12 and older provided their own consent in addition to parental or guardian consent.

### Recruitment

Respondents were recruited via the research team’s network, professional associations, social media, SLT students, and direct outreach to practices. SLTs that contributed to the recruitment of at least 15 respondents were able to receive credits for the Dutch quality register for SLTs. Gift vouchers of 10€ were raffled among all respondents.

We aimed to recruit a minimum of 300 respondents, but preferably 500 respondents [32, 33]. According to COSMIN guidelines, this sample size would be rated as ‘doubtful’ (250–499 participants) [34]. However, due to challenges in data collection, we initially assessed whether analyses could be reliably conducted with 300 respondents (verified by testing unidimensionality). These preliminary checks indicated that the psychometric analyses, including reliability and construct validity, could be adequately performed. Simulation studies further support this approach, showing that for unidimensional polytomous models, a sample size of *N* = 300 is sufficient for stable and accurate parameter estimation [35].

### Data collection

Data was collected between September 2024 and July 2025. The standards of the COSMIN initiative were used [27]. These standards provide criteria for the design and evaluation of studies on measurement properties, including content validity, structural validity, construct validity, and reliability. In line with COSMIN guidance, we applied these criteria when planning the study design. We applied the specific COSMIN criteria for each measurement property in our statistical analyses.

Participants were recruited via a flyer containing a link and QR-code, directing them to the MyCommunciation-Children survey. This online survey, hosted on Crowdtech© 2024 (ISO 27001 and ISO 27701 certified), integrated informed consent, sociodemographic information, MyCommunication-Children and comparator questionnaires. Participants were instructed to complete the survey independently, although parents or speech and language therapists could offer support with reading or navigation.

Sociodemographic information collected included gender, age, school type (regular vs. special education), grade, multilingualism, and type of communication problem (self-reported, participants could select one or more categories: language problems, speech problems, voice problems, hearing loss, or “other, namely…”). Objective diagnostic assessments (e.g., standardized language testing or cognitive assessment) were not conducted because the aim was to develop a generic instrument for communicative participation, for which children’s subjective experiences were central.

### Questionnaire battery

The MyCommunication-Children questionnaire and three comparator instruments were administered to assess construct validity. These comparators included: (1) a questionnaire for participation; (2) a single item measuring self-perceived communication; and (3) a single item measuring communicative participation.

### MyCommunication-children

MyCommunication-Children is a 49-item pool developed through literature review and qualitative research [28–30], designed to measure communicative participation from the perspective of children with speech, language, voice disorders, or hearing loss. Children from a young age were actively involved in the concept elicitation phase (aged 5–13; *n* = 13) as well as in studies evaluating content validity (aged 5–12; *n* = 30). Based on their direct input, item wording, response options, and the overall questionnaire format were iteratively refined to ensure comprehensibility and relevance for younger children [29, 30]. Items address multiple participation subdomains linked to the ICF Activities and Participation chapter [36]. The questionnaire uses a 4-point scale ranging from 4 (no difficulty) to 1 (cannot do), with three items including an additional NA option for content less relevant to some children [30].

### PROMIS^®^ Pediatric Peer Relationships short form [37]

The PROMIS v2.0 Peer Relationships short form 8a [38] was used to assess social participation and the quality of peer relationships. Validated in Dutch by the Dutch-Flemish PROMIS group [37, 39], it has demonstrated structural and construct validity and reliability. The validation study indicated a largely unidimensional structure with one item misfit, expected correlations with related constructs (*r* = 0.61 with PedsQL Social Functioning), minimal cross-cultural DIF, and reliable scores for most respondents (87.7% full item bank; 81.6% short form; 82.7% CAT) [37]. The short form includes 8 items with 5-point response options (1; never to 5; always), and higher T-scores reflect higher levels of social participation.

### Single item communication

Due to the absence of a validated instrument for children’s self-perceived communication, a single-item measure was developed: “Communication means talking, reading, understanding, writing, or otherwise making oneself understood. How much difficulty do you have with communication?” Responses use a 4-point scale: 4 (no difficulty) to 1 (much difficulty). Scores were expressed as sum scores, with high scores indicating less difficulty in communication.

This item was developed specifically for this study and has not yet been validated. It was included to provide a global self-rating of communication and to support the evaluation of construct validity.

### Single item communicative participation

Similarly, no suitable instrument existed for measuring children’s communicative participation, leading to the creation of a single-item measure: “Communication occurs in many situations, for example at home, school, with friends, or family. How well can you participate in situations where you need to communicate?” Responses use the same 4-point scale as the single item for communication. Scores were expressed as sum scores, with high scores indicating less difficulty in communicative participation.

This item was developed for the purpose of this study, and has not been validated. It was included to provide a global self-rating of communicative participation and to support construct validity analyses.

### Control question for possible help obtained

Finally, a control question assessed whether respondents received help during questionnaire administration (e.g., no help, help reading or help reading and choosing answers). This variable was used descriptively, with the possibility of differential item functioning analyses in later work if sample size permits.

### Survey administration

The survey could be completed on any device (phone, tablet, computer etc.). All items in the questionnaire battery were shown one by one, as recommended by the *Guidelines for communication-friendly questionnaires* [40]. All items were mandatory, except for the three items with an NA option. The survey consisted of multiple versions, each with its own fixed order of items. Responses were saved automatically, participants could pause and continue the survey at aby time without losing their answers. The average time of completion was 18 min.

### Data analysis

In line with COSMIN recommendations, this study focused on the evaluation of structural validity, construct validity, and reliability. Content validity was evaluated in a separate study and therefore not reassessed here [30]. Structural validity was examined through CFA, EFA, bifactor modeling, and IRT assumptions testing. Construct validity was evaluated by testing three a priori hypotheses regarding expected correlations with related constructs. Reliability was assessed using IRT-based standard errors and marginal reliability.

Data were analyzed following the PROMIS Psychometric Evaluation and Calibration Plan [41] using RStudio (v2025.05.1) [42]. Analyses were conducted by EA and NW (both SLTs and PhD candidates) and BS (IRT expert).

Our analysis strategy was to restrict the item pool to a unidimensional construct: communicative participation. The decision to target a unidimensional construct was grounded in the conceptualization of communicative participation as described in the Introduction. This choice is consistent with earlier psychometric research showing that related item banks (e.g., CPIB, MyCommunication-Adults) exhibit a predominantly unidimensional structure [16, 43]. The item bank initially contained 49 items with some overlap, allowing removal of poorly performing items without compromising content validity [30].

### Preliminary item analyses

Poorly functioning items were removed and smaller factors eliminated to identify a unidimensional set. The R-package *Psych* was used to conduct these analyses (V2.5.3) [44]. The frequencies of the four response options were analyzed. Items with response options selected only once or twice were removed.

Hereafter, the item-total correlations (corrected for unreliability) were examined within the domains of the ICF Activities and Participation chapter. Items with an item-total correlation less than 0.4 were removed. Items with an item-total correlation higher than 0.8 were considered indicative of possible duplication, but were not yet removed.

Correlations across the different ICF domains in the instrument were examined to assess whether the subdomains were conceptually related. Although items covered multiple ICF subdomains (‘self-care’, ‘domestic life’, ‘interpersonal interactions’, ‘major life areas’, ‘community/social life’), these primarily ensured content validity rather than indicating multidimensionality [43]. We therefore expected that the ICF subdomains, although different in content, would correlate with each other. This was checked by examining correlations between the domains. Mean item scores were calculated for each subdomain of the ICF Activities and Participation model, and Pearson correlations between these mean scores were computed. A correlation coefficient of 0.7 or higher was considered indicative of sufficient conceptual overlap. Some subdomains included only a limited number of items. Where appropriate, we merged subdomains to ensure sufficient coverage.

Final in the preliminary analysis, inter-item correlations were calculated using polychoric correlations to examine possible duplicate items. Correlations higher than 0.7 were considered to be significantly high [45]. In the decision of possible removal of items, EA and NW consulted the qualitative information from the content validity study [30], and selected the most relevant and comprehensible item. In addition, we aimed for an average inter-item correlation between 0.15 and 0.50 [46, 47].

After removal of poor functioning items in the preliminary item analysis, we assessed the item pool on structural validity in which we worked towards unidimensionality.

### Structural validity

To assess the structural validity of MyCommunication-Children, a Graded Response Model (GRM; a model designed for ordinal response categories, that allows item discrimination to vary across items) [48] was fitted to the data. The GRM model requires unidimensionality (the assumption that all items reflect a single underlying construct), local independence (the assumption that no residual associations remain between items after accounting for the latent trait), and monotonicity (the requirement that the probability of endorsing higher response categories increases as the level of the latent trait increases).

Unidimensionality was examined using confirmatory and exploratory factor analyses (CFA and EFA) via R package *Lavaan* (v0.6-19) [49]. CFA evaluates how well the factor model represents the observed data using several fit indices. The fit indices included comparative fit index (CFI > 0.95 for good fit), Tucker-Lewis Index (TLI > 0.95 for good fit), root mean square error of approximation (RMSEA < 0.06 for good fit), and standardized root mean residuals (SRMR < 0.08 for good fit) [41]. EFA included examining a screeplot, parallel analysis and McDonald’s bifactor model [41]. Parallel analysis was used to determine the number of meaningful factors by comparing the observed eigenvalues with the expected values based on chance. McDonald’s bifactor model provided two key indices: Omega Hierarchical (OmegaH) estimates the proportion of total score variance that is explained by the general factor, and explained common variance (ECV), which reflects the proportion of shared variance explained by the general factor. We tested the possibility of multiple factors, and assessed whether additional items needed to be removed to work towards a unidimensional scale. Evidence for unidimensionality included: first factor explaining ≥ 20% of variance, eigenvalue ratio > 4, standardized loadings on the general factor > 0.50, explained common variance (ECV > 0.60), and Omega Hierarchical (OmegaH > 0.70) [50]. Items with loadings < 0.50 were considered for removal.

Local dependence (LD) was assessed by looking at the residual correlations in the EFA model. Items with residual correlations higher than 0.2 were considered for deletion [41]. We assessed their impact on the discrimination parameter by temporarily removing one of the items in an item pair with correlations > 0.2. If local dependence was present, notable changes in the discrimination parameters would be expected.

Monotonicity was assessed using Mokken scaling (R package *Mokken*, v3.1.2) [51, 52]. In case the items with the NA option were still in the item pool, these items were temporally removed for the assessment of monotonicity. Monotonicity was assessed for all items individually (Hi values ≥ 0.30) and the H value of the entire scale (H value ≥ 0.50 for good monotonicity, H value of ≥ 0.40 for moderate monotonicity) [53].

If all the assumptions were met, a GRM was fitted to estimate the item discrimination and difficulty parameters, using R-package *mirt* (v. 1.44.0) [54], with the default Bock-Aitkin EM algorithm [55]. To assess item fit, the S-X2 statistic was used and p-values < 0.001 were considered indicative of misfit [56].

### Construct validity

Based on prior research on communicative participation, three hypotheses were formulated and tested to evaluate the construct validity of MyCommunication-Children [43]. Since communicative participation is the overlap between the constructs *communication* and *participation* we expected (1) a high correlation between MyCommunication-Children and the single item for communicative participation (> 0.7); (2) a moderate correlation between MyCommunication-Children and the single item for communication (0.4–0.6); (3) a moderate correlation between MyCommunication-Children and the PROMIS Peer Relationships (0.4–0.6) [2, 43]. Construct validity was tested for the entire item bank, and for the short forms.

### Development short forms MyCommunication-Children

Based on the output, two short forms were developed. The first short form aimed to cover the entire item bank. This process was informed by the following criteria: (1) discrimination parameter preferably > 1.5; (2) selected items reflect comprehensiveness of the content, in which at least all 5 domains of the ICF model were covered; (3) variation of difficulty thresholds across the theta scale.

A second short form was developed, specifically containing items for communicative participation in school setting. For this short form we set the following criteria: (1) discrimination parameter preferably > 1.5; (2) selected items cover content that is about communicative participation in school context; (3) variation of difficulty thresholds across the theta scale.

### Reliability

Reliability of the item bank and short forms was assessed by the proportion of respondents with standard errors (SE) ≤ 0.32 (reliability ≥ 0.90) and ≤ 0.447 (reliability ≥ 0.80) [57]. Marginal reliability curves were plotted to visualize T-scores exceeding these thresholds.

## Results

A total of 310 respondents completed the questionnaire battery. Most reported problems with language (*n* = 224), followed by speech (*n* = 88), voice (*n* = 29), hearing loss (*n* = 21), and other communication difficulties (*n* = 15). Mean scores were 2.9 for communication, 3.1 for communicative participation (both SD = 0.68), and 48.6 for PROMIS Peer Relationships (SD = 7.0). Participant characteristics are shown in Table 1.

**Table 1:** Participant characteristics

Sociodemographic	*N* participants
Mean age 4–6 7–10 11–13	8.9 years (SD 1.86) 11 231 64
Gender Female Male Other	142 167 1
Type of school Regular school Special education	256 54
Multilinguism No Yes	172 138
Type of communication problem Voice Speech Language Hearing Other	29 88 224 21 15
Help needed?	
No help needed	83
Help reading needed	112
Help reading and answering items	115

**Table 2:** Final item bank MyCommunication-Children, including item text

Item nr.	Item	Frequency of endorsement (%)			
		Cannot do (1)	Much difficulty (2)	A little difficulty (3)	No difficulty (4)
CPY5.5	I can tell the doctor about my symptoms.	7.4	15.8	42.6	34.2
CPY6.2	I can answer questions from the cashier.	6.8	12.3	41.0	40.0
CPY6.3	I can ask a store employee something.	7.1	12.3	38.7	41.9
CPY6.5	I can have a conversation with the hairdresser.*	4.5	4.2	31.6	44.5
CPY7.1	I can tell my parents about my day.	1.6	7.4	34.8	56.1
CPY7.3	I can have a conversation with family during dinner.	3.2	7.1	29.4	60.3
CPY7.4	I can have a conversation with my brother or sister.*	1.6	4.5	21.3	63.2
CPY7.5	I can have a conversation with my parents	1.0	2.9	30.3	65.8
CPY7.7	I can give my opinion to my parents.	2.3	5.5	29.0	63.2
CPY7.15	I can talk to a group of friends.	1.9	9.4	40.3	48.4
CPY7.16	I can talk to friends while playing sports, playing, or gaming.	2.3	7.1	35.2	55.5
CPY7.19	I can start a conversation with someone I want to be friends with.	3.5	13.5	42.9	40.0
CPY7.20	I can have a conversation with a friend.	1.3	2.6	32.9	63.2
CPY7.21	I can resolve a conflict with a friend.	6.8	21.3	46.1	25.8
CPY7.26	I can ask other children something.	2.3	6.8	39.0	51.9
CPY7.27	I can have a conversation with children my age.	1.6	7.4	35.5	55.5
CPY7.28	I can ask a group of children if I can play along.	3.9	10.3	36.8	49.0
CPY7.31	I can have a conversation with someone I don’t know.	11.9	25.8	43.9	18.4
CPY7.34	I can ask a stranger for directions.	15.2	20.3	37.1	27.4
CPY7.36	I can introduce myself.	2.3	10.0	25.8	61.9
CPY8.8	I can participate in a conversation in class.	2.9	10.0	46.8	40.3
CPY8.13	I can talk to classmates during the break.	2.3	3.5	29.4	64.8
CPY8.23	I can discuss with classmates during a group assignment.	2.6	12.3	40.3	44.8
CPY8.25	I can ask classmates questions.	1.6	5.5	36.5	56.5
CPY8.27	I can tell the teacher something when I am sad or angry	6.5	19.0	40.3	34.2
CPY8.31	I can have a conversation with a new child at school.	4.2	12.6	42.9	40.3
CPY8.32	I can give a presentation to the class.	13.9	25.2	38.4	22.6
CPY8.34	I can tell the teacher something.	1.0	7.4	35.8	55.8
CPY8.36	I can answer the teacher’s questions.	1.0	9.7	44.8	44.5
CPY8.38	I can understand the explanation of the gymnastics teacher.	1.3	2.9	27.4	68.4
CPY9.5	I can have a conversation with friends at a party.	2.3	7.4	30.0	60.3
CPY9.7	I can have a conversation with family at a party.	2.3	5.5	32.9	59.4

*CPY6.5 15.2% NA; CPY7.4 9.4% NA

Note. Items are listed in English, but this is unofficially translated using AI

### Preliminary item analysis

In seven out of 49 items, one of the response option “cannot do” was used only once or twice. These items were removed. For two items, the response category NA was used more than 10% (talking to hairdresser and a crush). These items were flagged, but not yet removed.

Item-total correlations indicated two items below 0.40 (talking to a crush, calling in case of emergency), which were removed. Two items with very high item-total correlations (> 0.80) were flagged but retained for further testing.

Correlations between subdomains led to the merging of “self-care” and “domestic life,” resulting in three subdomains overall. Correlations between subdomains were all > 0.70 (see Fig. 1 in Supplementary Materials 1).

**Fig. 1:**
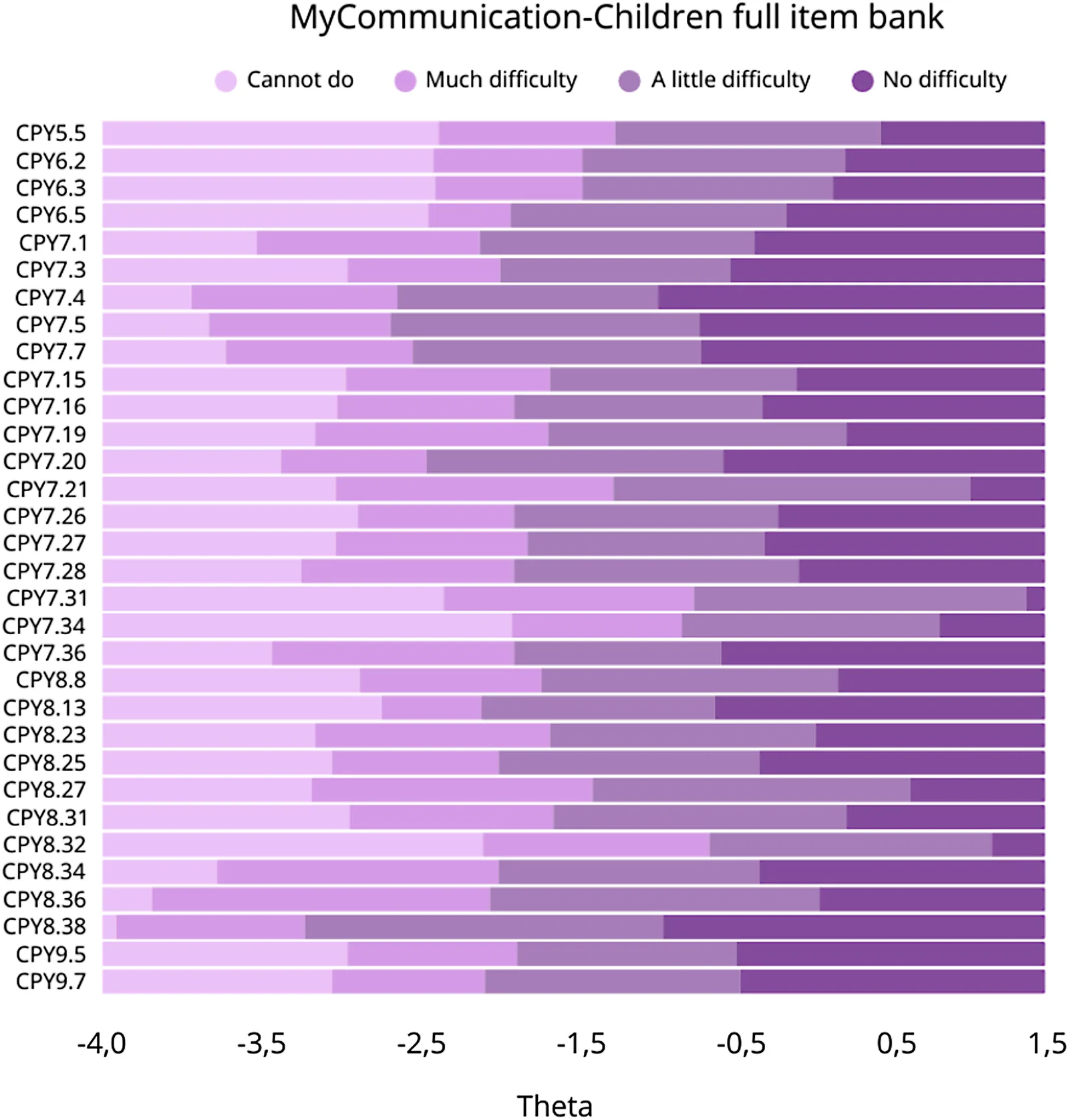
Location of the response category thresholds of the full item bank along the theta scale (θ). Thresholds are the points at which the probability of selecting a higher response category is equal to the probability of selecting an lower category

**Fig. 2:**
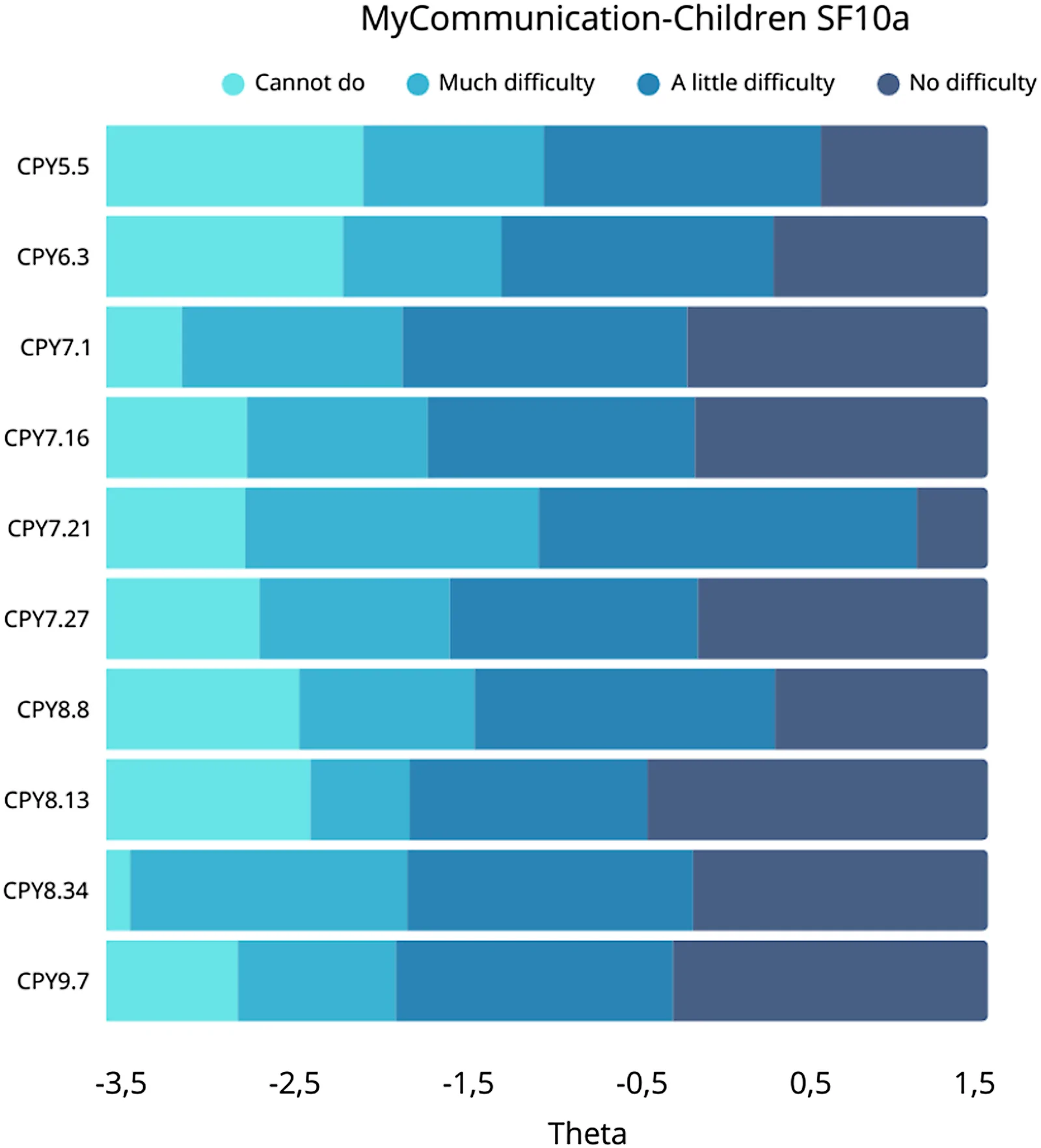
Location of the response category thresholds of the SF10a along the theta scale (θ). Thresholds are the points at which the probability of selecting a higher response category is equal to the probability of selecting an lower category

**Fig. 3:**
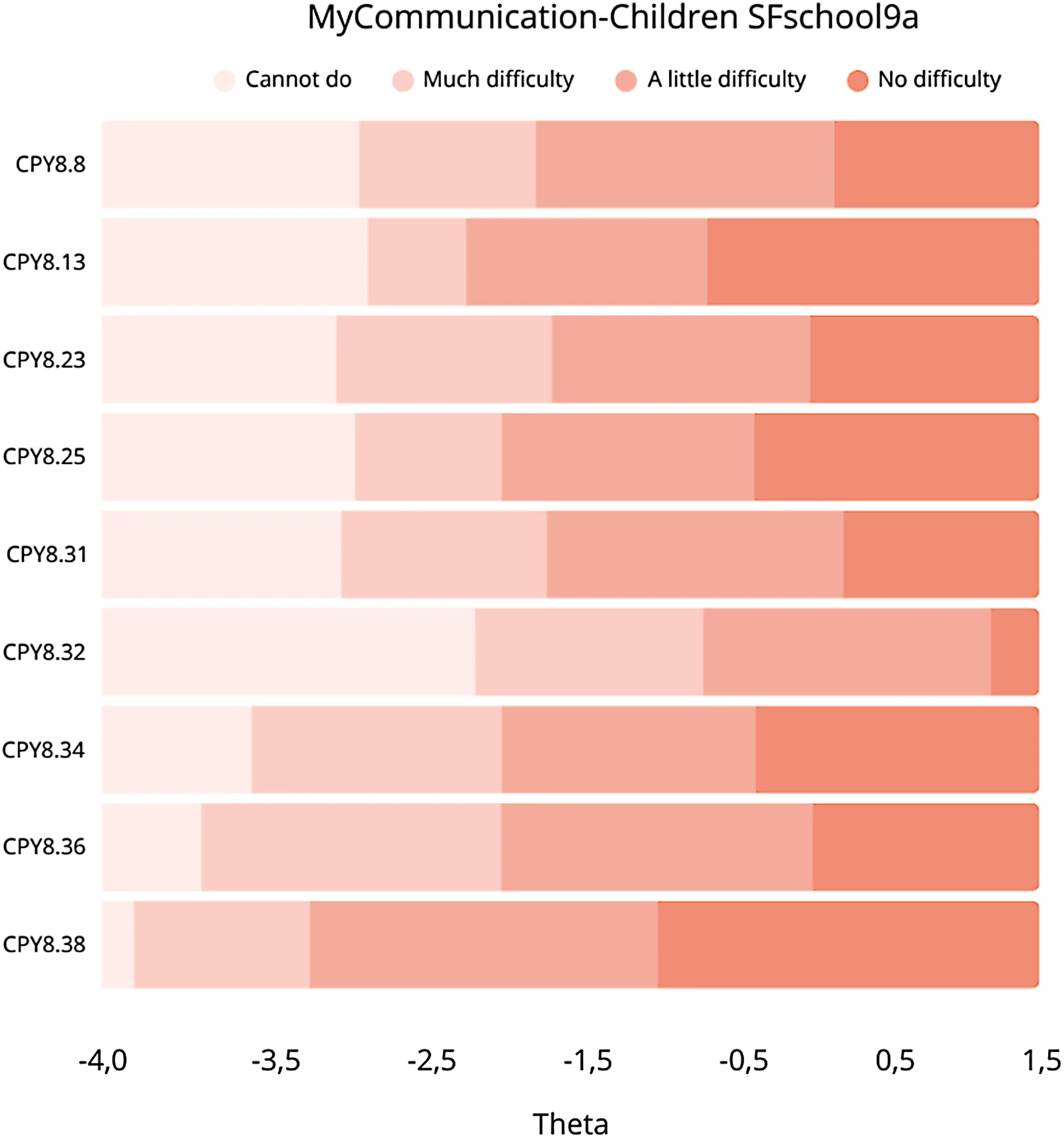
Location of the response category thresholds of the SFschool9a along the theta scale (θ). Thresholds are the points at which the probability of selecting a higher response category is equal to the probability of selecting an lower category

One item was removed due to high inter-item correlation (0.82) with a similar item (“understanding the gymnastics teacher’s assignment” vs. “understanding the teacher’s explanation,” the latter retained). Within the subdomains, many inter-item correlations were low (< 0.4). The average inter item correlation between all items was 0.38.

**Fig. 4:**
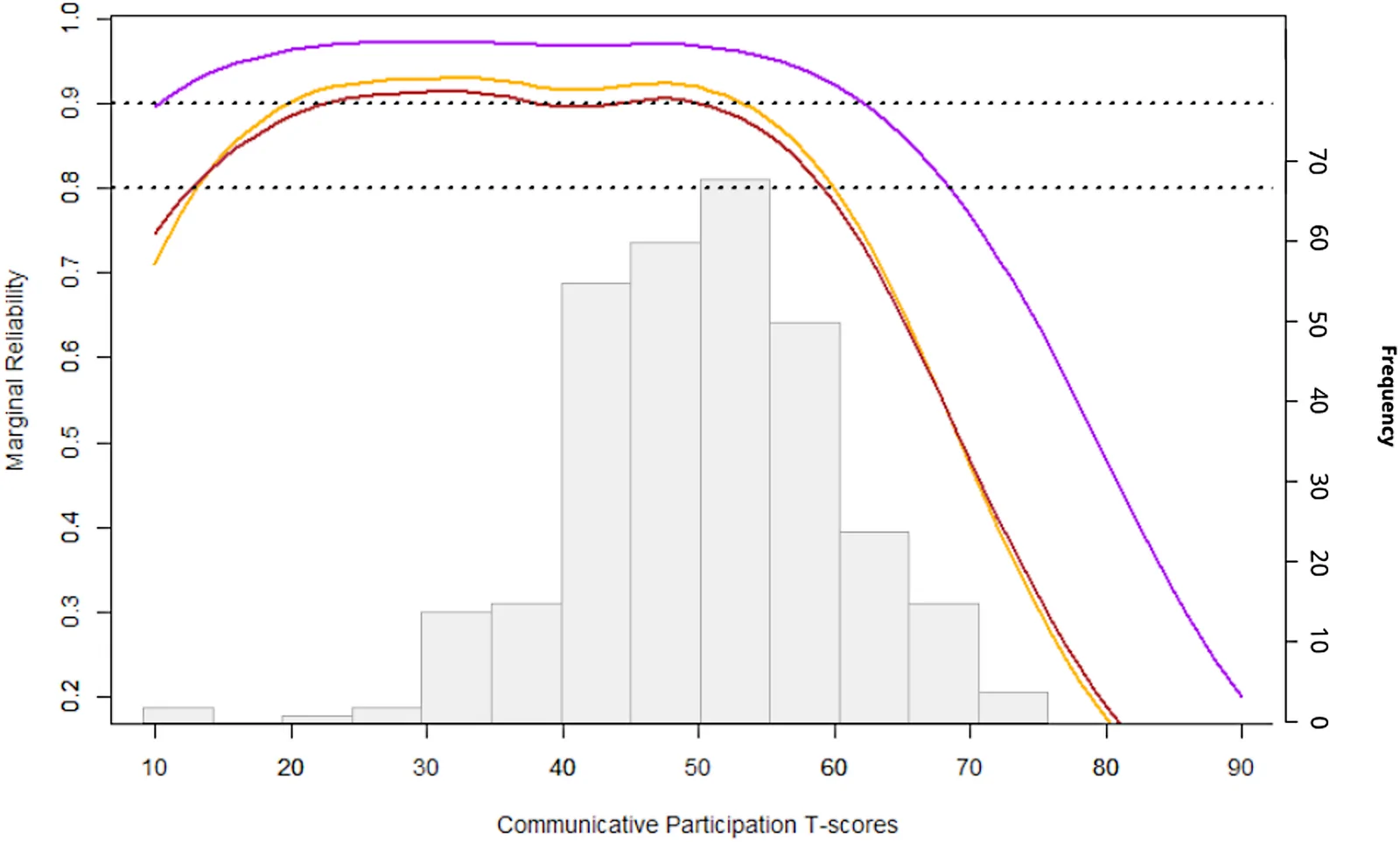
Marginal reliability across the latent score range for the full item bank (purple), the SF10a (yellow), and the SFschool9a (brown) of the MyCommunication–Children measure. Grey bars show the sample distribution of T-scores. Horizontal dashed lines indicate reliability levels of 0.80 and 0.90

In total, 10 items were removed during preliminary analysis, leaving 39 items for further validation. Full documentation of analyses, item removal, and rationale is provided in Supplementary Material 2.

### Structural validity

A confirmatory factor analysis (CFA) showed good (unscaled) fit statistics (CFI 0.987; TLI 0.986; SRMR 0.071; RMSEA 0.067), supporting sufficient unidimensionality. Parallel analysis revealed eight eigenvalues > 1, with a dominant first factor (15.7 vs. 2.0; ratio 7.85:1), suggesting a dominant general factor. McDonald’s bifactor model showed an OmegaH of 0.88 and ECV of 0.80, indicating sufficient unidimensionality. Most items loadings were sufficiently strong on the general factor. A one-factor EFA model showed two items with low factor loadings (< 0.50), and were removed. One possible subfactor was identified, containing items with loadings of 0.4–0.5. These items were about talking with parents or siblings. Item loadings on the general factor were at least 0.1 higher compared to this subfactor. Based on all above factor analyses, the item pool was considered unidimensional enough to continue analysis.

Of the 666 item pairs, seven showed residual correlations > 0.20, indicating local dependence. Three items were removed due to overlap; four pairs were retained based on unique content. Their impact was tested by temporarily removing and reintroducing items, showing no meaningful effect on discrimination parameters. These items were therefore retained (see Supplementary Material 2).

To assess monotonicity, two items were temporarily removed because of the NA option. H values showed moderate monotonicity (H = 0.45, and H_i_ > 0.30 for all items).

The remaining 34 items were modeled with a Graded Response Model (GRM). No item misfit was detected with S-X2. Items with low discrimination (< 1.5) were reviewed; two items (“I can tell my parents that I don’t feel good” and “I can tell the teacher that I feel sick”) were removed due to limited relevance.

The final CFA on the 32-item bank again demonstrated excellent fit (CFI = 0.993; TLI = 0.993; SRMR = 0.062; RMSEA = 0.053). The standard errors of the estimated IRT parameters were generally low to moderate: for the discrimination parameter (*a*), the mean SE was 0.22 (median 0.21, range 0.15–0.32), and for the three difficulty parameters, mean SEs were 0.31, 0.17, and 0.11, respectively (medians 0.28, 0.15, 0.10; ranges 0.19–0.56, 0.12–0.28, 0.08–0.19). Table 2 lists the final items and response distributions, while Fig. 1 illustrates the location of response thresholds across the theta (θ) scale.

### Construct validity

The 32-item MyCommunication-Children showed moderate correlations with related measures. Correlations were *r* = 0.50 with the single-item communication measure, *r* = 0.54 with the single-item communicative participation measure, and *r* = 0.48 with the PROMIS Peer Relationships short form.

### Development of short form MyCommunication-Children

Two short forms were derived from the item bank. The first, a general short form (SF10a), covers a broad range of communicative participation and consists of 10 items. Figure 2 shows the location of response thresholds for each item along the theta (θ) scale.

A second short form focused specifically on communicative participation in the school context (SFschool9a) and consists of 9 items. Figure 3 presents the location of response thresholds for these items along the θ scale.

CFA on the both short forms showed good fit, with the TLI slightly high (SF10a: CFI = 0.985; TLI = 0.980; SRMR = 0.042; RMSEA = 0.063; SFschool9a: CFI = 0.977; TLI = 0.969; SRMR = 0.048; RMSEA = 0.083). The SF10a showed moderate correlations with the comparator measures (*r* = 0.48 with the single-item communication, *r* = 0.53 with the single-item for communicative participation, and *r* = 0.44 with the PROMIS Peer Relationships short form). The SFschool9a showed moderate correlations as well (*r* = 0.51 with the single-item communication, *r* = 0.55 with the single-item for communicative participation, and *r* = 0.48 with the PROMIS Peer Relationships short form).

### Reliability

The full 32-item bank demonstrated strong measurement precision, with a standard error (SE) ≤ 0.316 for 90% of respondents and ≤ 0.447 for 97%. The general short form (SF10a) achieved SE ≤ 0.447 for 84% of respondents, and the school short form (SFschool9a) reached the same threshold for 84%.

Figure 4 displays marginal reliability across the range of Communicative Participation T-scores for the full item bank (purple), the general short form (yellow), and the school short form (brown). The full bank provided the highest reliability (> 0.90) across a wide score range, while both short forms maintained high reliability in the central score range where most respondents were located (see grey histogram). Reliability of the short forms declined more quickly at the lower and upper extremes of the scale. For the X-axis theta scores were converted to T-scores (theta*10 + 50).

### Post-hoc analysis of assistance during completion of MyCommunication-Children

Based on the control item, 115 of the 310 children reported having received assistance with answering the questions. Because this help may have influenced the IRT parameters, we conducted post-hoc differential item functioning analyses (DIF). DIF is useful to detect biased responding for items, given group membership, such as sex or age [41]. Although this sample size was below the recommended threshold for robust DIF analyses, exploratory DIF analyses were conducted, using R package *Lordif* (v 0.4.2) [58]. First, children who completed the questionnaire independently and children who received assistance with reading only were collapsed into a single group (*n* = 195), as reading assistance was permitted and children were instructed to select their responses independently. This group was compared with children who reported receiving assistance with answering the questions (*n* = 115). DIF detection was based on the Chi-square criterion. Effect sizes were evaluated using McFadden’s pseudo-R², with R² ≥ 0.02 indicating meaningful DIF [58]. No evidence of DIF was found between children who reported receiving assistance with answering and those who did not.

To further explore the potential influence of assistance, a second analysis compared children who completed the questionnaire independently (*n* = 83) with all children who received any form of assistance (*n* = 227). One item showed DIF (about understanding the gymnastics teacher; R² = 0.0339), but without a consistent theoretically explanation.

To explore potential group differences at the scale level, we compared T-scores for the two groups. Children who completed the questionnaire independently or with reading assistance scored significantly higher (M = 51.88, SD = 8.65) than children who received help with answering (M = 46.71, SD = 10.40), t(197.49) = 4.45, *p* < 0.001.

These findings suggest that although the items function equivalently across groups, children receiving assistance with answering demonstrate lower levels of communicative participation.

The amount of help children reported when completing the questionnaire battery varied by age. Among the youngest children (4–6 years), most (81.8%) needed assistance with both reading and answering, whereas older children (7–10 and 11–13 years) were more often able to complete the questionnaire independently, though a substantial proportion still required support with reading and answering (38.5% and 20.3%, respectively). Additional exploratory DIF analysis compared children aged 7–9 with those aged 10–13 years, and identified one item with potential DIF (about asking a question to a store employee; R² = 0.0244). This finding can be theoretically explained, as this behavior is more typical for older children.

When analyzed by type of communication problem, there were few clear differences. Children with voice problems were more likely to report that they needed no help (48.3%) compared to children with other types of problems (20.1%-40.9%), but overall, the distribution of assistance required was similar across the different types of communication difficulties. Detailed results can be found in Supplementary Materials 1.

## Discussion

This paper aimed to assess the psychometric properties of MyCommunication-Children, a newly developed item bank that measures communicative participation in children with communication problems. Structural validity, construct validity and reliability were assessed. Seventeen of 49 items were removed during preliminary analysis to optimize psychometric robustness. Factor analyses supported unidimensionality of communicative participation. Moderate correlations with related measures (general communication, communicative participation, and peer relationships) supported construct validity. Two short forms were constructed with a different purpose: one measuring communicative participation in all contexts (10-item short form) and one measuring communicative participation in the school context (9-item short form). Reliability analyses suggest that the item bank provides high measurement precision across a broad range of scores, with the full item bank outperforming the short forms, which nonetheless maintained acceptable reliability within the central range of the scale. However, given the relatively small and heterogeneous sample, these findings should be interpreted with this in mind, and warrant further replication in larger samples.

### Preliminary item analysis

Seventeen items were removed from the original pool based on statistical and conceptual considerations. These items are listed in the Supplementary Materials 2. Items with extremely low endorsement of the response category “cannot do” were excluded to prevent sparse data issues, as recommended by Reeve [41]. While statistically justified, this may also indicate ceiling effects or limited relevance for the target population. Many of these excluded items reflected basic communicative acts (e.g., understanding parents or teachers, expressing simple needs), which appeared too easy and showed little variability.

Beyond low endorsement, additional psychometric criteria guided item removal. Specifically, two items were removed due to low item-total correlations, one item due to low inter-item correlation, two items with low factor loadings in exploratory factor analysis, three items showing local dependence, and two items with low discrimination parameters. Several patterns emerged from these removals that go beyond purely statistical considerations. Some items were not yet fully relevant for the target population, for example “talking with a crush” or “calling in case of an emergency,” indicating that these situations may not be universally experienced by children in this context. Items that are easy for most children (e.g., reporting feeling sick or understanding a teacher’s explanation) showed low discrimination parameters. Additionally, certain items showed conceptual overlap, consistent with expectations from the development phase, such as “talking with the doctor” and “telling the doctor about my symptoms,” resulting in local dependence. Overall, the removal of these items reflects a combination of psychometric properties, context-specific relevance, and conceptual redundancy.

The item pool initially included three items with a NA response option, reflecting findings from the content validity study [30] that certain communicative situations (e.g., talking to a hairdresser, talking with a crush, talking with siblings) were not relevant to all children. In the present analysis, two of these items were marked NA by more than 10% of respondents. “Talking with a crush” was removed due to low inter-item correlations, while “talking to a hairdresser” was retained with the NA option because of its unique content, despite not being selected for the short form. This approach underscores the importance of context-sensitive item development. The small number of NA responses overall suggests that instrument relevance was not compromised, but rather enhanced by sensitivity to individual differences in life experiences.

Finally in the preliminary analysis, relationships between the qualitative subdomains, based on ICF activities and participation chapters, were examined. These subdomains were not expected to represent distinct psychometric dimensions but instead to ensure comprehensive coverage of communicative participation. Correlations confirmed that the subdomains did not reflect separate latent traits, but rather different contextual expressions of a single broad construct.

### Communicative participation as unidimensional construct

Confirmatory factor analysis (CFA) showed good fit indices, supporting the assumption of unidimensionality. However, given ongoing debates about the limitations of CFA fit indices [59], additional analyses were conducted. Parallel analysis suggested multiple factors, but the first-to-second eigenvalue ratio (7.85:1) indicated a dominant general factor. McDonald’s bifactor model further supported this, with OmegaH and ECV showing the general factor accounted for most common variance. A potential subfactor (items on communication with parents or siblings) loaded more strongly on the general factor (minimum difference 0.10), justifying a unidimensional model. The average inter-item correlation (0.38) reflects conceptual breadth rather than multidimensionality.

These results are consistent with studies of adult instruments based on the same theoretical framework. For example, the CPIB [16] and My Communication-Adults [43] also demonstrated sufficient unidimensionality despite initial indications of multiple factors, with local dependence addressed through item refinement. This consistency across different age-populations supports the robustness of communicative participation as a single overarching construct. It aligns with the ICF model, suggesting that communicative participation can be conceptualized as a unified construct encompassing multiple contexts and types of interactions.

Monotonicity analysis showed a moderate scalability coefficient (H = 0.45), meeting Mokken criteria [51, 53]. While higher H would ensure stronger ordering, the current level was sufficient for IRT modeling, with conceptual diversity likely explaining the moderate monotonicity.

### Construct validity

Three hypotheses were tested to evaluate construct validity. Two were confirmed: moderate correlations were found between MyCommunication-Children and the single-item communication measure (*r* = 0.50) and the PROMIS Peer Relationships short form (*r* = 0.48). The expected high correlation with the single-item communicative participation measure was not observed (*r* = 0.54). According to COSMIN, construct validity is sufficient if ≥ 75% of hypotheses are confirmed [27]; in this study, 67% were supported. One possible explanation for this correlation is the nature of the comparison measure: the single item for communicative participation was developed specifically for this study and has not been validated. The absence of a validated gold standard for communicative participation in children represents a limitation in the current study. Therefore, conclusions regarding construct validity should be interpreted with caution.

### MyCommunication-Children as instrument

In addition to the full 32-item bank, two short forms were developed to facilitate practical use in clinical and educational settings. The 32-item bank was designed to provide comprehensive coverage of communicative participation, ensuring that all relevant facets of the construct are represented. Because the item bank is calibrated using modern psychometric methods, it allows for flexibility in administration: not all items need to be administered to obtain reliable scores. This flexibility enables the creation of short forms tailored to specific purposes.

Both short forms demonstrated acceptable reliability. The first, a 10-item general short form, covers a wide range of communicative contexts (e.g., interactions with health-care professionals, parents, peers, family, store employees, classmates, and teachers). Its diversity makes it a suitable and comprehensive tool for clinical practice. The second, a 9-item school-focused short form, targets communicative participation in educational settings. This short form was developed with the purpose of supporting the process of decision-making in school regarding the need for additional support in the classroom, or serve as one component of the assessment process for eligibility for specialized educational services. By selecting items most relevant to the school context and omitting items that are less applicable for this specific purpose (e.g. items from other ICF-domains), the school short form enhances content validity for the school context and practical utility. While the short forms demonstrated adequate reliability, its use should be accompanied by careful interpretation and complemented by clinical judgement and additional sources of information. Future research should examine how both short forms are used and perceived by speech and language therapists (SLTs), and whether their application improves identification and support of children with communication problems.

MyCommunication-Children was developed to capture the perspectives of children themselves. However, during data collection, some participants received assistance. Two forms of help were reported. First, some children received help reading the items. This type of assistance was anticipated and allowed, as it was provided in the content validity study as well [30]. However, even this form of assisted self-report comes with a risk of bias: research has shown that when children are assisted, they may be influenced (consciously or unconsciously) by the presence of an adult, potentially leading to socially desirable responses [60–63]. While this represents a limitation of the study, it appeared necessary to ensure accessibility for children who would otherwise be excluded from participation [30].

Second, and more concerning, is that some children were also assisted in selecting their response options. Both younger and older children frequently received this type of help, and no clear differences were observed across types of communication problems. Notably, during the content validity study, children were able to choose their answers independently [30], indicating that such assistance was not strictly necessary. Although it is unknown what type of assistance was actually provided, which is a limitation of the study, instructions clearly stated that children were supposed to choose their answers independently. It is unclear why assistance in responding was provided. It is possible that some children were unable to select responses independently, in which assistance may have enabled participation that would otherwise not have been possible. Alternatively, children may have been capable of answering independently, but adults may have been insufficiently accustomed to positioning children as primary informants of their own experiences. From a child centered care perspective, this latter interpretation is concerning and highlights the need for greater awareness in the role of children as active respondents.

Comparisons of T scores showed that children who completed the questionnaire independently or with reading assistance scored higher than those who received help with selecting their answers. This suggests that assisted responding is associated with lower levels of communicative participation. Assistance may therefore reflect underlying differences in children’s communicative functioning or in the circumstances under which assistance is provided.

To further examine whether assistance influenced the measurement properties of the instrument, exploratory Differential Item Functioning (DIF) analyses were conducted. These analyses did not reveal substantial evidence of DIF. Although these findings should be interpreted cautiously due to the limited sample size, they provide a preliminary indication that assistance with answering did not systematically bias item responses at the scale level.

At the same time, the absence of meaningful DIF does not resolve the broader conceptual and ethical concerns associated with assisted responding. Even if measurement properties appear unaffected, assistance with answering may still compromise the authenticity of the child’s perspective and runs counter to the fundamental aim of MyCommunication-Children to elicit children’s own perspectives. Therefore, findings from the present study should be interpreted with caution, and the use of the short forms in clinical practice should be considered supportive rather than determinative, particularly in contexts involving high-stakes decision making.

These findings underscore the importance of establishing and communicating clear guidelines for the administration of the item bank. Future research should further investigate the conditions under which assistance is provided and examine its impact on measurement properties in larger samples.

Within this broader context of ongoing instrument development, this study describes the first assessment of the psychometric properties of MyCommunication-Children. The instrument is part of a larger measurement system, MyCommunication-Youth, that contains versions for adolescents and young adults as well. Although we have developed one scale for children, it would be valuable to obtain scales for other age groups as well, and link the scores between the three instruments (if possible four, including MyCommunication-Adults [43].

### Age of participants

In this study, we initially aimed to include children from seven years of age. However, speech and language therapists were deliberately given the option to include slightly younger children if, based on clinical observation, they judged that the child had sufficient reflective capacity to answer questions. This option was used eleven times. In doing so, we aimed to be as inclusive as possible and to capture the child’s own perspective, even if some support was required to answer questions, as we considered this preferable to excluding the child entirely. Common practice in PROM research often sets a fixed lower age limit of eight years. Our approach differs by allowing some flexibility. At the same time, there are contrasting perspectives in the literature: for example, Varni et al. [64] showed that children as young as five years can meaningfully self-report on their health, and other authors emphasize that the minimum age cannot be strictly defined, as developmental trajectories vary widely between children [65, 66]. A scoping review by Gale & Carlton [67] found that only a limited number of studies included children under eight in cognitive interviews, but that participation is possible when methods are adapted to the child’s developmental level. Furthermore, Gale et al. [68] demonstrated that children aged six to seven can meaningfully participate in cognitive interviews when the methodology is developmentally adapted, whereas PROM developers in practice generally include children only from eight years onward, largely due to the absence of clear guidance [69]. Our approach, allowing experienced therapists discretion in inclusion, aligns with these findings.

Importantly, our approach is also consistent with international guidance on pediatric PRO administration. The International Society for Pharmacoeconomics and Outcomes Research (ISPOR) task force on pediatric PROs explicitly states that younger children (approximately 4–7 years) often require interviewer-administered formats or parental assistance due to limited reading ability and attention span [65]. In this age group, completing a measure with adult support is considered an appropriate and commonly used administration method, provided that the child remails the source of the response.

At the same time, we recognize the methodological implications. Children younger than seven years are a potentially vulnerable subgroup, particularly in the presence of language developmental disorders. This topic will therefore be one that requires more research. In future studies, it will be important to analyze this youngest age group separately. In the current study, however, the numbers were too small to allow such an analysis.

## Limitations

This study contained several limitations. First, the sample size was relatively small, with only 310 respondents completing the questionnaire battery. Although this number was sufficient for analysis, the sample size may have influenced item removal. For example seven items that had low frequencies on the response option “cannot do”. In addition, due to the small sample size we were not able to perform additional analysis, such as differential item functioning (DIF). Future research should assess DIF for factors like gender, type of communication disorder, multilingualism, education type, and (again) assistance provided during assessment.

Second, within the sample that we included, the majority of participants reported having language difficulties, such as language delay or developmental language disorder. While this reflects the primary client population of SLTs and aligns with the intended focus of the instrument, it may limit the generalizability of the findings to other populations with communication difficulties, such as those with speech and voice disorders or hearing loss. Future studies should include more diverse populations to confirm reliability and validity across communicative profiles.

Third, no validated self-reported instruments specifically measuring communicative participation in children were available, so a single self-constructed item was used to assess construct validity. This item was not tested for comprehensibility or relevance with the target population, limiting the strength of conclusions.

Fourth, a proportion of children received adult assistance during questionnaire completion, including assistance with reading items and, in some cases, selecting response options. Although this issue is discussed in detail elsewhere in the manuscript, it represents an important limitation. Exploratory DIF analyses did not indicate systematic differences between children who received assistance with answering and those who did not, but findings remain preliminary due to limited sample sizes and heterogeneity in the type and extent of assistance. Consequently, the potential impact of assisted responding on item functioning and parameter estimates cannot be definitively determined.

## Conclusions and implications

This study provides initial psychometric evidence for the validity and reliability of MyCommunication-Children, an instrument that measures communicative participation from the perspective of children with communication difficulties. The two short forms derived in this study offer promising options for fixed-length administration and may facilitate efficient assessment of communicative participation across contexts. The results have practical, research, and policy implications, while also highlighting the need for further research: clinicians can assess participation across contexts and plan interventions efficiently; researchers can use the instrument as an outcome measure and explore cross-cultural applicability; and policymakers may consider such standardized, evidence-based tools in effort to support inclusion and guide programs for children with communication disorders, emphasizing participation alongside communicative development.

## Supplementary Information

Below is the link to the electronic supplementary material.

Supplementary Material 1

Supplementary Material 2

## Data Availability

Data will be made available on reasonable request.
