# Supplementary material for: Psychometric properties of MyCommunication-Children: an item bank for measuring communicative participation in children with speech, language, voice disorders or hearing loss
Source: J Patient Rep Outcomes. 2026 Jun 17;10:156. doi: 10.1186/s41687-026-01118-w (PMC13518642; doi:10.1186/s41687-026-01118-w)
Supplement: Supplementary file 1 [file 41687_2026_1118_MOESM1_ESM.docx]

**Supplementary Materials 1**

**Figure 1**
*Correlations across different subdomains of MyCommunication-Children, based on the initial item pool (49 items)*

Note: Correlations between different subdomains of MyCommunication-Children, based on the initial item pool (49 items). The figure displays correlations among the subdomains “unknown people,” “ICF domain 7,” and “domain school.” Higher correlation values indicate stronger associations between subdomains.

**Figure 2**
*Expected Total Score for MyCommunication-Children, final item set (32 items)*

*Note:* Expected total score on MyCommunication–Children (final item set, 32 items) as a function of underlying communicative ability (θ), estimated using an item response theory model. The X-axis represents the child’s estimated ability level, while the Y-axis shows the expected total score. The curve illustrates that scores increase continuously with ability.

**Figure 3**
*Item probability functions of MyCommunication-Children, final item set (32 items)*

*Note:* Item characteristic curves for the final 32 items of MyCommunication-Children. Each curve represents the probability of selecting a particular response category as a function of the child’s estimated ability (θ).

**Figure 4**
*Item information curves of MyCommunication-Children, final item set (32 items)*

*Note:* Item information curves for the final 32 items of MyCommunication-Children. Each curve represents the amount of statistical information an item provides across the underlying ability scale (θ), estimated from the item response theory model. The X-axis represents the child’s ability level, and the Y-axis represents item information. Higher peaks indicate that the item is most precise at that ability level, while lower values indicate less precision.

**Figure 5**
*Test information and standard errors of MyCommunication-Children, final item set (32 items)*

*Note*: Test information curve (TIC) and corresponding standard errors for the MyCommunication–Children instrument (32 items). The X-axis represents the child’s estimated ability level (θ), while the Y-axis shows the amount of test information (left axis) and the standard error of measurement (right axis). Higher test information indicates greater precision in estimating ability at that level, whereas lower standard errors correspond to higher measurement reliability. The figure demonstrates that the instrument provides the most precise measurement for children with low to medium ability levels.

**Figure 6**
*Screeplot analysis of MyCommunication-Children, based on the larger item pool (39 items)*

*Note:* Screeplot of eigenvalues from the factor analysis of the MyCommunication-Children item pool (39 items). The X-axis represents the factor number, and the Y-axis shows the corresponding eigenvalues. The steep drop in eigenvalues after the first factor suggests that a single dominant factor accounts for most of the variance, indicating a largely unidimensional structure.

**Table 1.** Overview of reported help during administration of MyCommunication-Children, split in age groups.

| **Age group** | **No help needed (n, %)** | **Help reading (n, %)** | **Help reading and answering (n, %)** |
| --- | --- | --- | --- |
| **4–6 years** | 1 (9.1%) | 1 (9.1%) | 9 (81.8%) |
| **7–10 years** | 50 (21.6%) | 92 (39.8%) | 89 (38.5%) |
| **11–13 years** | 30 (46.9%) | 21 (32.8%) | 13 (20.3%) |

**Table 2.** Overview of reported help during administration of MyCommunication-Children, split in type of communication problem**.**

| **Type communication problem** | **No help needed (n, %)** | **Help reading (n, %)** | **Help reading and answering (n, %)** |
| --- | --- | --- | --- |
| **Voice** | 14 (48.3%) | 10 (34.5%) | 5 (17.2%) |
| **Speech** | 36 (40.9%) | 23 (26.1%) | 29 (33.0%) |
| **Language** | 45 (20.1%) | 95 (42.4%) | 84 (37.5%) |
| **Hearing** | 7 (33.3%) | 7 (33.3%) | 7 (33.3%) |
| **Other** | 2 (13.3%) | 6 (40.0%) | 7 (46.7%) |
